# Surface Properties of Global Land Surface Microwave Emissivity Derived from FY-3D/MWRI Measurements

**DOI:** 10.3390/s23125534

**Published:** 2023-06-13

**Authors:** Ronghan Xu, Zharong Pan, Yang Han, Wei Zheng, Shengli Wu

**Affiliations:** 1 Key Laboratory of Radiometric Calibration and Validation for Environmental Satellites, National Satellite Meteorological Center (National Center for Space Weather), China Meteorological Administration, Beijing 100081, China; 2 Innovation Center for FengYun Meteorological Satellite (FYSIC), Beijing 100081, China; 3 General Institute of Water Resources and Hydropower Planning and Design, Ministry of Water Resources, Beijing 100120, China; 4 Earth System Modeling and Prediction Center, China Meteorological Administration, Beijing 100081, China

**Keywords:** brightness temperature, microwave radiation imager, land surface microwave emissivity, remote sensing, land cover types

## Abstract

Land surface microwave emissivity is crucial to the accurate retrieval of surface and atmospheric parameters and the assimilation of microwave data into numerical models over land. The microwave radiation imager (MWRI) sensors aboard on Chinese FengYun-3 (FY-3) series satellites provide valuable measurements for the derivation of global microwave physical parameters. In this study, an approximated microwave radiation transfer equation was used to estimate land surface emissivity from MWRI by using brightness temperature observations along with corresponding land and atmospheric properties obtained from ERA-Interim reanalysis data. Surface microwave emissivity at the 10.65, 18.7, 23.8, 36.5, and 89 GHz vertical and horizontal polarizations was derived. Then, the global spatial distribution and spectrum characteristics of emissivity over different land cover types were investigated. The seasonal variations of emissivity for different surface properties were presented. Furthermore, the error source was also discussed in our emissivity derivation. The results showed that the estimated emissivity was able to capture the major large-scale features and contains a wealth of information regarding soil moisture and vegetation density. The emissivity increased with the increase in frequency. The smaller surface roughness and increased scattering effect may result in low emissivity. Desert regions showed high emissivity microwave polarization difference index (MPDI) values, which suggested the high contrast between vertical and horizontal microwave signals in this region. The emissivity of the deciduous needleleaf forest in summer was almost the greatest among different land cover types. There was a sharp decrease in the emissivity at 89 GHz in the winter, possibly due to the influence of deciduous leaves and snowfall. The land surface temperature, the radio-frequency interference, and the high-frequency channel under cloudy conditions may be the main error sources in this retrieval. This work showed the potential capabilities of providing continuous and comprehensive global surface microwave emissivity from FY-3 series satellites for a better understanding of its spatiotemporal variability and underlying processes.

## 1. Introduction

The surface emissivity at microwave frequencies plays an important role in the retrieval of atmospheric and surface parameters, the study of vegetation phenology, the understanding of climate trends, land surface and sub-surface processes, and the assimilation of satellite data in numerical weather prediction (NWP) models [1,2,3,4,5,6,7]. The emissivity is the ratio of surface-emitting radiation to blackbody-emitting radiation, which reflects the surface thermal radiation characteristics. The primary intended application of microwave emissivity is to provide surface emissivity constraints in atmospheric and surface property retrieval, to serve as a dynamic indicator of land surface properties relevant to climate change monitoring, and to be a crucial boundary condition that affects NWP systems in improving reliable global medium-range weather forecasts [8,9]. Presently, compared with the atmospheric parameters, the surface emissivity spectra at microwave frequencies over land are less understood. Due to the challenges of distinguishing changes in land surface properties such as soil types, vegetation canopy, topography, soil moisture, and temperature, microwave emissivity over land is highly variable in spatial and temporal terms compared with that over the ocean [8,10].

Microwave land emissivity can be broadly derived based on field observation experiments, a physical microwave emissivity model, and retrieval from satellite observation. Field observation experiments were carried out by ground-based and airborne microwave radiometers. This method can obtain typical land types emissivity with limited area and specific experimental conditions.

Microwave emissivity models have been developed based on the experimental results and physical principles of microwave emissivity. Although the uncertainty in simulating microwave land emissivity is still a major obstacle in NWP systems, the advantage of the model simulation method is that there is a physical constraint placed upon the retrievals, where the various parameters being retrieved must be consistent with one another. A surface emissivity model has been developed and successfully integrated into the community radiative transfer model (CRTM) at the US Joint Center for Satellite Data Assimilation [11,12]. This model can be used to determine land emissivity under various surface conditions. For surfaces such as snow, desert, and vegetation, the volumetric scattering from the medium was included and first calculated, and the brightness temperatures at the top of the medium were derived from a two-stream radiative transfer approximation. The CRTM was used as the forward operator in the NOAA/microwave integrated retrieval system (MIRS) to perform the retrieval in all-weather conditions [13]. The radiative transfer for television infrared observation satellite (TIROS) operational vertical sounder, RTTOV, was also developed for simulating satellite radiances at variable wavelengths [14]. Microwave emissivity models require the input of a large number of surface parameters, such as surface type, soil moisture, vegetation characteristics, and surface roughness. Therefore, it is difficult to obtain a large scale of microwave emissivity with a lack of an accurate surface property database.

In recent years, satellite observation has become the main method to obtain large-scale regional and global microwave emissivity. A space-borne passive microwave (PMW) radiometer plays an important role in satellite observations to provide information on surface emissivity. It is necessary to correct the effect of surface emissivity from the PMW signals in order to retrieve the atmospheric parameters over the land, such as atmospheric temperature, humidity, cloud water content, and precipitation. Substantial progress has been made in retrieving microwave emissivity from passive microwave radiometers.

Direct observational-based algorithms use satellite brightness temperature (Tb) observations along with corresponding land and atmospheric properties to retrieve microwave emissivity. The basic idea of this method is the atmospheric radiation transfer equation and eliminating the radiation contribution of atmosphere, cloud, rain, and surface temperature with auxiliary data such as atmospheric temperature, water vapor profile, and surface temperature. This type of retrieval algorithm is supposed to be computationally easier and more reliable than land surface model-based retrieval [15,16]. There have been various techniques developed to derive the microwave emissivity over land using PMW measurements, such as the special sensor microwave imager (SSM/I) [17], the advanced microwave scanning radiometer for Earth observing System (AMSR-E) [18], the advanced microwave scanning radiometer 2 (AMSR2) [7], and the tropical rainfall measuring mission (TRMM) microwave imager (TMI). For example, the global maps of monthly mean land surface emissivity were derived from SSM/I data and ancillary cloud and land surface temperature products from the International Satellite Cloud Climatology Project (ISCCP) [19] and atmospheric data from NWP models. The AMSR-E data was used to develop land surface microwave emissivity with ancillary land surface temperature data from the moderate resolution imaging spectroradiometer (MODIS) on the same Aqua spacecraft, as well as water vapor and temperature profiles from global NWP models [8].

Moreover, microwave land emissivity can be retrieved by statistical and neural network methods with the relationship between emissivity and brightness temperature, as well as polarization ratio, microwave vegetation index, and soil wetness index [20]. This kind of method tries to construct parameters that are sensitive to land surface microwave emissivity and relatively insensitive to surface temperature and atmospheric parameters, so as to establish a statistical relationship between the constructed parameter and microwave emissivity.

The passive microwave radiometers such as SSM/I, SSMIS, AMSR-E, and AMSR2 were counted for the previous time series land surface microwave emissivity dataset. The status of these passive microwave sensors means there is a real risk of a gap in the passive microwave land surface microwave emissivity time series, which would likely compromise future research [21]. The continuous, consistent, and decadal dataset observed by the microwave radiation imager (MWRI) onboard the FengYun-3 satellite series provides valuable and sustained information on surface emissivity spectra over different surface types for numerical weather prediction model data assimilation [22]. The calibration bias difference of MWRI between ascending and descending orbits has been cross-corrected by the physical-based correction algorithm to improve data assimilation in the numerical weather predictions and reanalysis systems [23,24]. FengYun-3 satellite series, including FY-3B, FY-3C, and FY-3D, have accumulated PMW radiometer measurements for a long time, making China the only nation maintaining continuous passive microwave data from 2010 to today. The FY-3D satellite is the fourth satellite of the Chinese secondary generation FY-3 series and was launched on 15 November 2017 with the goal of observing global atmospheric and geophysical features. The observations from MWRI can be used to monitor environmental information such as the spatial and temporal distributions of sea ice, soil moisture, precipitation, clouds, and water vapor content in the atmosphere [25,26,27,28]. The next MWRI sensor is planned to be aboard the FY-3F platform, which will be launched in 2023. Therefore, the MWRI has great potential for continuous global land surface microwave emissivity collection to avert the looming gap [29].

On one hand, the objective of this study is to derive microwave emissivity of the land surface directly from FY-3D/MWRI observations and to further the legacy of the FY-3 mission in microwave land surface parameters. On the other hand, the objective is to characterize land surface microwave emissivity variations from different surface properties. In this study, the land surface microwave emissivities from 10.65 to 89 GHz are determined with the atmospheric and surface data from the ERA-Interim reanalysis data by removing the cloud and rain affection. The characteristics and error sources of the estimated microwave emissivity for different land cover types are also discussed.

## 2. Data and Methods

### 2.1. Data Sources

#### 2.1.1. Passive Microwave Remote Sensing Datasets

The MWRI loaded in the FY-3 satellite is a dual-polarized passive microwave radiometer and provides twice-daily measurements of the global microwave emissions over land with descending/ascending orbital equatorial crossings at about 1:30–13:30 local solar time. The MWRI instrument conically scans the Earth with a swath width of 1400 km. It observes the Earth’s surface in 10 microwave channels at the frequencies of 10.65, 18.7, 23.8, 36.5, and 89 GHz with vertical and horizontal polarization. The channel configuration and viewing geometry of MWRI are very similar to the existing microwave imagers, such as AMSR2, SSM/I, and TMI, which provide comparable measurements and products as MWRI retrieval. The AMSR2 instrument onboard the global change observation mission-water (GCOM-1) satellite continues Aqua/AMSR-E observations aiming to study changes in water circulation. AMSR2, similar to its predecessor AMSR-E, has a conical scan mechanism and obtains data over a ~1450 km swath with a 55° incidence angle. The GMI instrument, the evolution of TMI on TRMM, is a dual-polarization, multi-channel, conical-scanning, passive microwave radiometer with frequent revisit times onboard the GPM (global precipitation measurement) satellite. The MWRI brightness temperature data (L1 product, 25 km) provided by the National Satellite Meteorological Center (http://satellite.nsmc.org.cn/portalsite/default.aspx (accessed on 15 August 2022)) are used as inputs in the land surface microwave emissivity retrieval algorithm. The main parameters of passive microwave remote sensing sensors, including MWRI, AMSR2, and GMI, are shown in Table 1.

#### 2.1.2. Ancillary Datasets

The European Center for Medium-Range Weather Forecasts Re-Analysis (ERA-Interim) products are used to generate global atmospheric profiles. These products are generated by the European Center for Medium-Range Weather Forecasts (ECMWF) and have been available since 1979 to the present day. ERA-Interim has demonstrated its usefulness in remote-sensing applications [30]. In this work, the global ERA-Interim air temperature, geopotential height, relative humidity profiles, and skin temperature at 0.75° longitude × 0.75° latitude spatial resolution every 6 h corresponding to 00:00, 06:00, 12:00, and 18:00 Coordinated Universal Time (UTC) are used.

**Figure 1:**
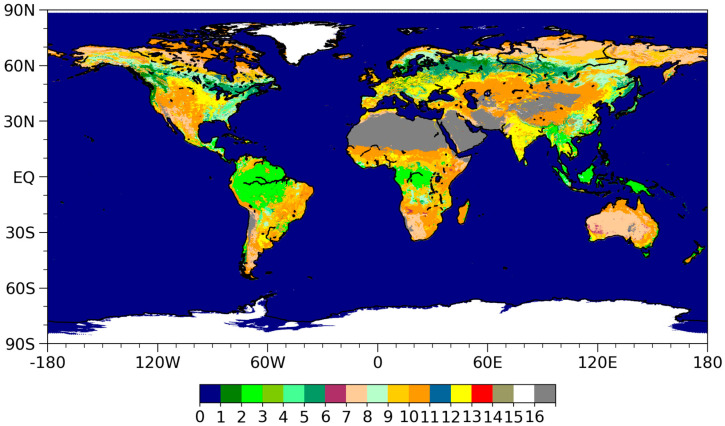
Global land cover data in IGBP classification system from MODIS product. Classification legends: 0-Water Bodies, 1-Evergreen Needleleaf Forests, 2-Evergreen Broadleaf Forests, 3-Deciduous Needleleaf Forests, 4-Deciduous Broadleaf Forests, 5-Mixed Forests, 6-Closed Shrublands, 7-Open Shrublands, 8-Woody Savannas, 9-Savannas, 10-Grasslands, 11-Permanent Wetlands, 12-Croplands, 13-Urban and Built-up Lands, 14-Cropland/Natural Vegetation Mosaics, 15-Permanent Snow and Ice, 16-Barren or Sparsely Vegetated.

The global 30-Arc-Second Elevation (GTOPO30) Digital Elevation Model (DEM) available from the U.S. Geological Survey (http://www.usgs.gov/centers/eros/science/usgs-eros-archive-digital-elevation-global-30-arc-second-elevation-gtopo30 (accessed on 20 July 2020)) is also used to amend the effect of the altitude of each pixel. Static surface type data, following the International Geosphere Biosphere Programme (IGBP) classification system, and the yearly land cover climate modeling grid MCD12C1 Version 6 data in a 0.05 degree range are taken from MODIS products (http://lpdaac.usgs.gov/products/mcd12c1v006 (accessed on 20 July 2020)). These data were resampled to 0.25 degrees by determining the majority value in a grid, as shown in Figure 1.

### 2.2. Methodology for Surface Microwave Emissivity Retrieval

To retrieve surface microwave emissivity from MWRI measurements, an approximated radiation transfer equation is used. Assuming that the land surface is flat and specular and considering the atmosphere free from clouds and precipitation, the radiation emanating from a non-scattering plane parallel atmosphere is related to surface emissivity, *ε*, and surface temperature, $T_{s}$. Using the Rayleigh-Jeans approximation, the atmospheric radiation transfer equation is expressed by Norouzi et al. [31], Yang, and Weng [32]:

(1) $${Tb}_{\left(p,f\right)}=\varepsilon_{\left(p,f\right)}T_{s}\Gamma +T_{u}+\left(1-\varepsilon_{\left(p,f\right)}\right)(T_{d}+T_{sky}\Gamma )\Gamma$$

where Tb is the observed satellite brightness temperature at polarization, p (horizontal or vertical), and the required frequency, f. ε and $T_{s}$ are the land surface emissivity and skin temperature, respectively. $T_{u}$ and $T_{d}$ are the brightness temperatures associated with upwelling and downwelling radiation components, respectively, and Γ is the atmospheric transmittance.

On the right side of Equation (1), the first term is the surface-emitted radiation, which is further attenuated by the atmosphere. The second term is the upward radiation toward the satellite. The third term is the atmosphere’s downward radiation reflected by the surface via atmospheric attenuation and the cosmic background radiation (about 2.7 K) reflected by the surface via atmospheric attenuation.

Via the above atmospheric radiation transfer equation, microwave emissivity requires parameters such as surface temperature, atmospheric transmittance, and upwelling and downwelling radiation. Solving the above equation for emissivity, the instantaneous global microwave emissivity is computed by the following expression:

(2) $$\varepsilon_{\left(p,f\right)}=\frac{{Tb}_{\left(p,f\right)}-T_{u}-(T_{d}+T_{sky}e^{-\tau \left(H_{s},H\right)/\mu })e^{-\tau \left(H_{s},H\right)/\mu }}{e^{-\tau \left(H_{s},H\right)/\mu }[T_{s}-\left(T_{d}+T_{sky}e^{-\tau \left(H_{s},H\right)/\mu }\right)]}$$

Equation (2) has been widely used for the derivation of surface microwave emissivity. The retrieval can be addressed from a direct inversion of the radiation transfer equation, which requires detailed knowledge of the vertical structure of the atmosphere in order to account for atmospheric transmissivity and radiance (both upwelling and downwelling). Here, $H_{s}$ is altitude of the land area under studying above the sea level, and H is the upper boundary of the atmosphere. Usually, H=100 km. Respectively, $\tau_{s}$ is the optical thickness of the atmosphere from the sea level to the altitude $H_{s}$, $\tau_{H}$ is the same for the altitude H, and

(3) $$\tau (H_{s},H)=\tau_{H}-\tau_{s}$$

In fact, $\tau_{s}$ is the virtual parameter. Then, in the Rayleigh-Jeans approximation B(τ,T)≈T, and the upward radiation toward the satellite can be written as (4). B is the brightness temperature at each layer with optical depth τ; μ is related to the satellite view angle θ and μ=cos⁡(θ).

(4) $$T_{u}=\int_{\tau_{s}}^{\tau_{H}}T\;\;exp\left[-\frac{\left(\tau_{H}-\tau \right)}{\mu }\right]d\tau /\mu$$

The atmosphere’s downward radiation reflected by the surface via atmosphere attenuation can be written as (5).

(5) $$T_{d}=\int_{\tau_{s}}^{\tau_{H}}T\;\;exp\left[-\frac{\left({\tau -\tau }_{s}\right)}{\mu }\right]d\tau /\mu$$

$T_{u}$, $T_{d}$, and τ are computed from the temperature and water vapor profiles that are inputs to the microwave absorption model.

### 2.3. Detection of Precipitation Affected Radiances

For the brightness temperatures, especially those at frequencies higher than 10 GHz, the scattering effect is detected due to the existence of large rain, cloud liquid water, and ice particles in the atmosphere.

Since the scattering effect of raindrops on high-frequency microwaves will reduce the high-frequency brightness temperature, the difference between low- and high-frequency brightness temperatures is used for detection algorithms for precipitation detection [33]. For the accuracy of the retrieval of surface emissivity from satellite measurements, the difference between ${Tb}_{23.8v}$ and ${Tb}_{89v}$ is defined as a positive threshold to exclude those pixels affected by atmospheric scattering.

(6) $${Tb}_{23.8v}-{Tb}_{89v}>8K\;and\;{Tb}_{89v}>270K$$

When the brightness temperature difference is 8 K or greater and the brightness temperature at 89 GHz vertical polarization is 270 K or greater, the pixel is flagged as precipitation-affected radiance. Furthermore, according to the ERA-Interim data, the cloud liquid water greater than 0.05 kg/m^2^ was filtered to exclude rain conditions.

### 2.4. Data-Processing Flow

For land surface microwave emissivity retrieval from MWRI measurements, the observed satellite brightness temperature from 10.65 to 89 GHz at both vertical and horizontal polarization states was used to retrieve the surface emissivity.

The brightness temperature difference between low and high frequencies is used to produce screening flags to exclude those pixels affected by precipitation. High-frequency brightness temperatures and the LandSeaMask dataset from MWRI data were used to exclude the water bodies. The components of atmospheric radiation and atmospheric transmittance were calculated using the CRTM and the 6-h ERA-Interim datasets containing surface skin temperature, atmospheric temperature, and humidity profiles.

The surface temperature $T_{s}$ is obtained by assuming it is approximately the same as the skin temperature from the ERA-Interim datasets. The reanalysis datasets are interpolated to the local time of the FY-3D overpass and to the center of the MWRI footprints. The ERA-Interim analysis terrain elevations are adjusted to the GTOPO30, spatially averaged with MWRI footprint resolution, and atmospheric water vapor was corrected for the elevation difference.

**Figure 2:**
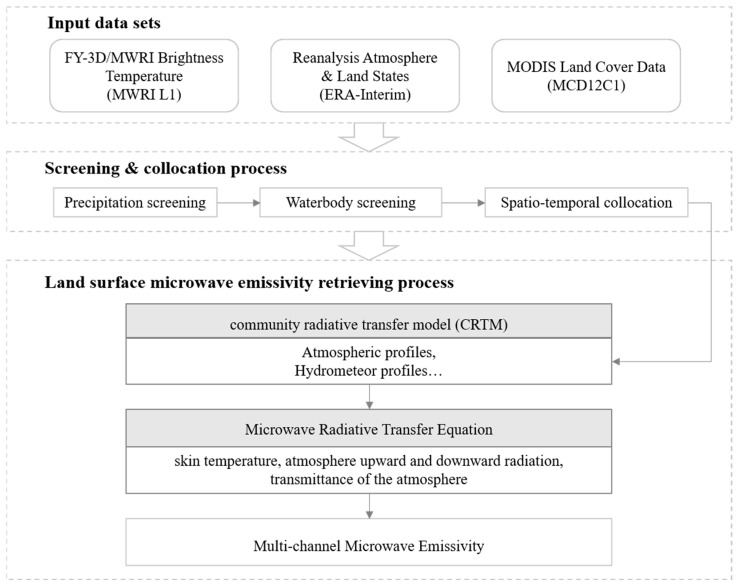
Data-processing flow of microwave land surface emissivity retrieval.

The instantaneous surface emissivity was averaged with the monthly composition. Then, the IGBP land cover data were regridded to the same locations as the MWRI emissivity, with spatial averaging to obtain the fraction of each grid point for each classification. The data-processing flow of land surface microwave emissivity derivation is shown in Figure 2.

## 3. Results and Discussion

### 3.1. Global Distribution of Land Surface Emissivity from MWRI

**Figure 3:**
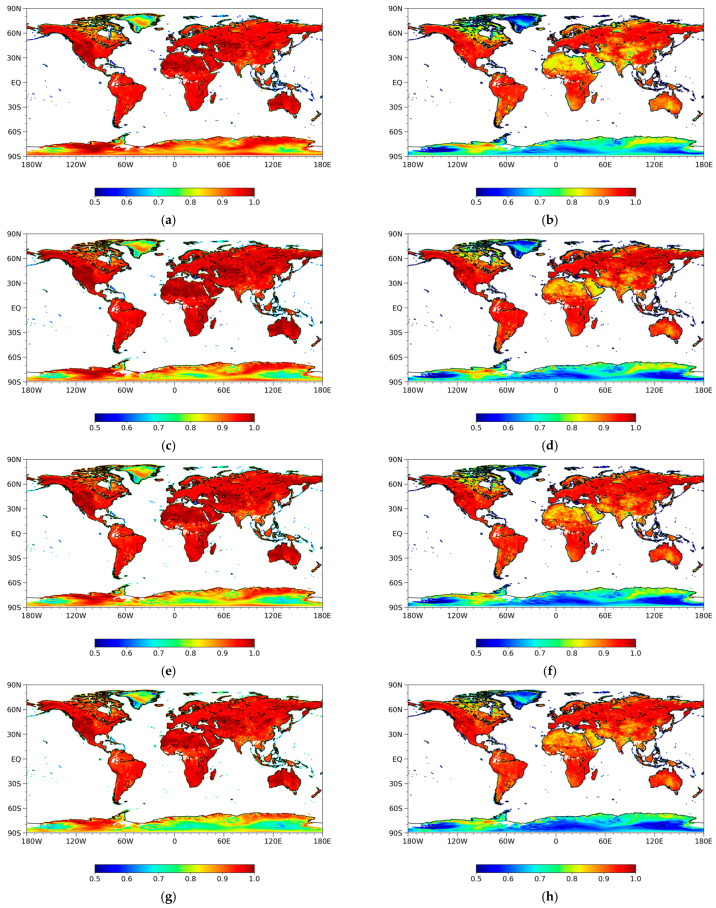

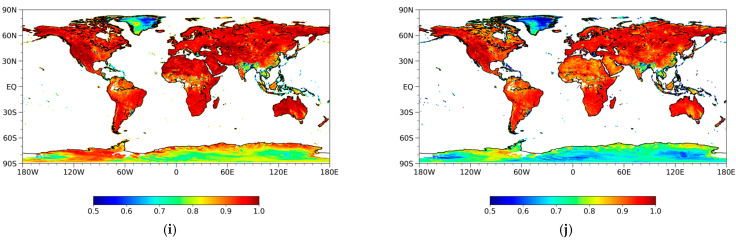
Land surface microwave emissivity for July 2018 from MWRI ascending observations at vertical (left) and horizontal (right) polarizations for the 10.65, 18.7, 23.8, 36.5, and 89 GHz channels (from top to down): (**a**) emissivity for 10.65 GHz vertical polarization; (**b**) emissivity for 10.65 GHz horizontal polarization; (**c**) emissivity for 18.7 GHz vertical polarization; (**d**) emissivity for 18.7 GHz horizontal polarization; (**e**) emissivity for 23.8 GHz vertical polarization; (**f**) emissivity for 23.8 GHz horizontal polarization; (**g**) emissivity for 36.5 GHz vertical polarization; (**h**) emissivity for 36.5 GHz horizontal polarization; (**i**) emissivity for 89 GHz vertical polarization; (**j**) emissivity for 89 GHz horizontal polarization.

The global distribution of emissivity is calculated to determine the relative differences in emissivity variation among the five channels. Figure 3 presents the monthly emissivity estimates from MWRI ascending observations at vertical and horizontal polarizations for the 10.65, 18.7, 23.8, 36.5, and 89 GHz channels from July 2018. The known large-scale emissivity features and their variations are apparently represented by the estimates. The global distribution of emissivity is dependent on frequency over different land cover types. Low horizontal emissivity areas are distributed in desert, ice, and snow areas, such as the Sahara Desert, Greenland, and Antarctic, due to smaller surface roughness and increased scattering effects at a certain incident angle compared to the vegetated regions. The vertical polarization emissivity is greater than the horizontal polarization at the same frequency. The horizontal emissivity shows more significant heterogeneity than the vertical emissivity. The higher-frequency channels show a larger magnitude of emissivity than the lower-frequency channels. In high-density vegetated areas, the emissivity from low frequency is greater than that from high frequency, which is also the basis of the microwave emissivity difference vegetation index. For China, the vertical emissivity is higher in the northwest, with a general value greater than 0.95, while it is relatively low in the southeast, ranging from 0.87 to 0.92. Most of the vertical emissivity values in the Qinghai-Tibet Plateau are above 0.9, while the range of the horizontal emissivity values is around 0.8. This is consistent with the results presented in [34].

### 3.2. Spectrum Characteristics of Emissivity over Different Land Cover Types

**Figure 4:**
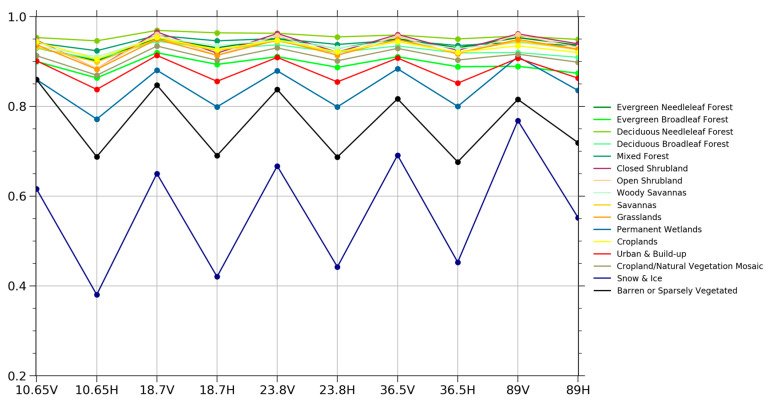
Emissivity variations at vertical and horizontal polarizations for different land cover types from 10.65, 18.7, 23.8, 36.5, and 89 GHz frequencies of MWRI in July 2018.

The variations of emissivity are investigated combined with different land cover types, as shown in Figure 4. The result shows that the vertical emissivity is greater than the horizontal emissivity at the same frequency. At the same time, the emissivity of the polarization difference decreases gradually as the frequency increases. The average emissivity in barren or sparsely vegetated areas is smaller than in vegetated areas. However, the difference between horizontal and vertical emissivity is higher in barren areas than in vegetation-covered areas. This is because the vegetation coverage is closely related to the surface roughness. The smaller the surface roughness, the closer to the specular reflection, the greater the polarization difference of the land surface emissivity [35]. With the gradual increase in surface roughness, the scattering effect in all directions increases, resulting in a gradual decrease in the polarization difference until it disappears. The polarization difference of emissivity is affected by the vegetation coverage, such as the forest size, type, and location. The emissivity of all vegetation is relatively stable, and its variation in vertical polarization is smaller than its variation in the horizontal polarization. Microwave emissivity is a function of vegetation structure, vegetation water content, and surface roughness, and all these factors have obvious seasonal variations.

Furthermore, the emissivity microwave polarization difference index (MPDI) for the 10.65 to 89 GHz channels is calculated for the comparison of different emissivity datasets, including the analysis of their sensitivity to the different land surface parameters. The MPDI can exhibit greater sensitivity to surface parameters and mitigate the effects of the atmosphere and land surface temperature. The MPDI has also shown that differences between horizontal and vertical polarization signals contain a wealth of information regarding soil moisture and vegetation density [36]. The emissivity-based MPDI is calculated as:

(7) $$MPDI=\frac{\varepsilon_{v}-\varepsilon_{h}}{\varepsilon_{v}+\varepsilon_{h}}$$

where $\varepsilon_{v}$ and $\varepsilon_{h}$ are emissivity at vertical and horizontal polarization, respectively, for a specific frequency.

**Figure 5:**
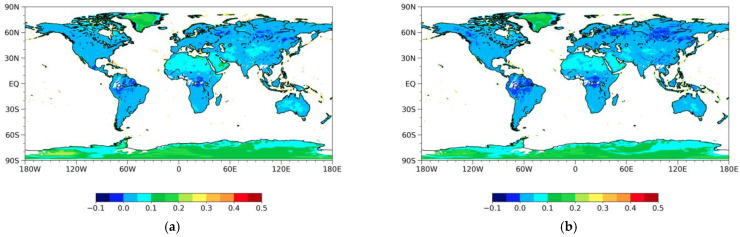

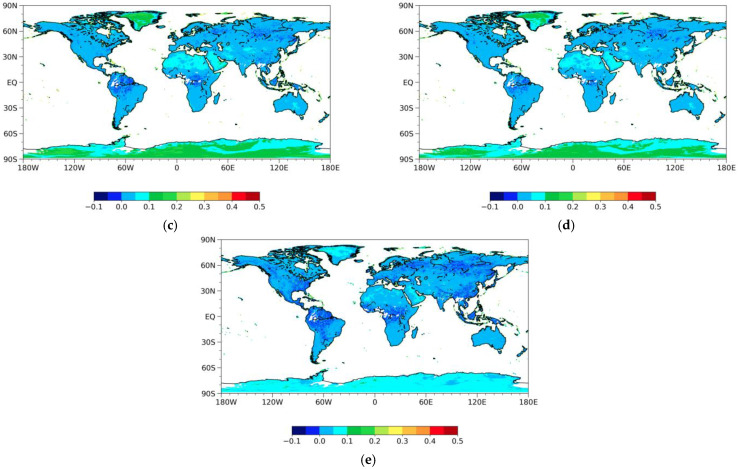
Emissivity microwave polarization difference index (MPDI) for July 2018 from MWRI ascending observations for the 10.65, 18.7, 23.8, 36.5, and 89 GHz channels (from top to down): (**a**) MPDI for 10.65 GHz; (**b**) MPDI for 18.7 GHz; (**c**) MPDI for 23.8 GHz; (**d**) MPDI for 36.5 GHz; (**e**) MPDI for 89 GHz.

Figure 5 shows the global distribution of MPDI calculated from MWRI. The emissivity estimates at horizontal and vertical polarizations do not necessarily show similar characteristics due to their different dielectric constant responses. Desert regions show high MPDI values ranging from about 0.05 to 0.15, which suggests the high contrast between vertical and horizontal microwave signals in this region. Therefore, from northwest to southeast China, the MPDI decreases gradually from about 0.15 to 0.

### 3.3. Seasonal Variations in Emissivity for Different Surface Properties

**Figure 6:**
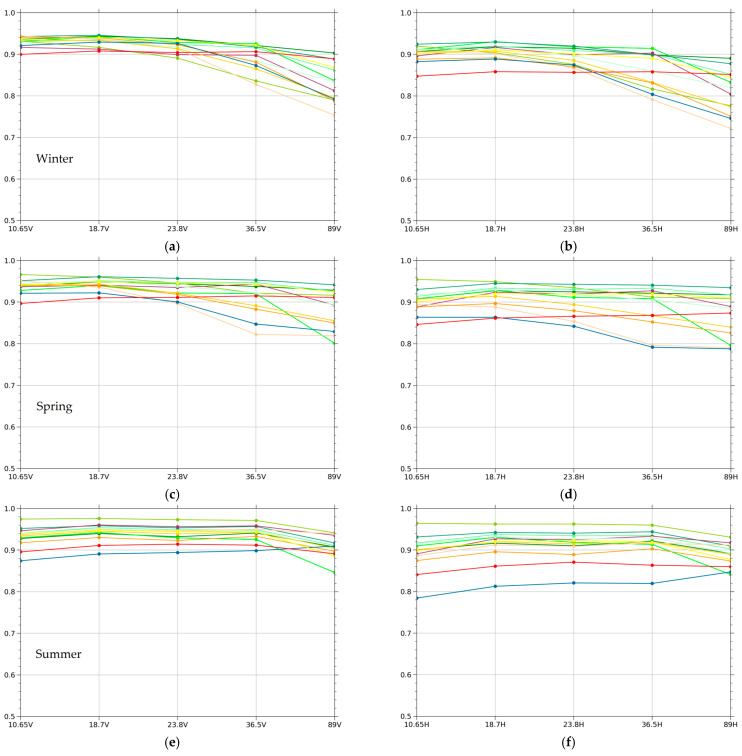

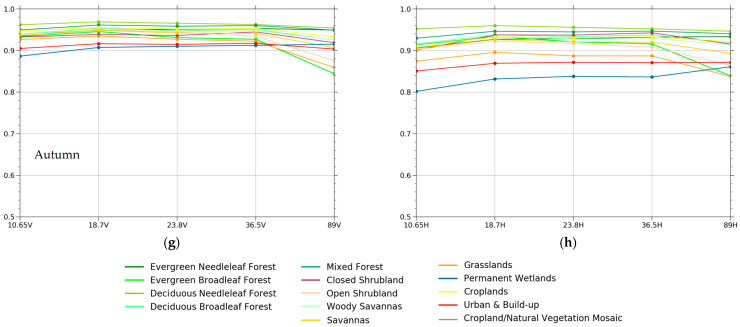
Seasonal emissivity variations at vertical (left) and horizontal (right) polarizations for different land cover types from 10.65, 18.7, 23.8, 36.5, and 89 GHz frequencies of MWRI: (**a**) winter variations in vertical emissivity; (**b**) winter variations in horizontal emissivity; (**c**) spring variations in vertical emissivity; (**d**) spring variations in horizontal emissivity; (**e**) summer variations in vertical emissivity; (**f**) summer variations in horizontal emissivity; (**g**) autumn variations in vertical emissivity; (**h**) autumn variations in horizontal emissivity.

The seasonal variations of emissivity are investigated in combination with different land cover types, as shown in Figure 6. We used January, April, July, and October as the representatives of spring, summer, autumn, and winter in the northern hemisphere. The seasonal variation characteristics of horizontal and vertical polarized emissivity for different land cover types, especially the vegetated region, are compared and analyzed. Overall, the variation characteristics of the summer and autumn seasons are consistent. In summer, the emissivity of deciduous needleleaf forests is relatively the highest among all frequencies, with an average horizontal polarization emissivity of 0.938 and an average vertical polarization emissivity of 0.951. Open shrublands have the lowest emissivity, with an average horizontal polarization emissivity of 0.885 and an average vertical polarization emissivity of 0.930. This might be due to the high vegetation densities in deciduous needleleaf forests and the relatively low vegetation densities in open shrublands. Emissivity increases with the increase in vegetation density. This is because, with the increase in vegetation density, surface roughness increases consistently. The average emissivity of deciduous needleleaf forests in summer is almost the highest among different land cover types. This may be related to the following three factors: vegetation density, vegetation water content, and vegetation composition structure. The vegetation density of the deciduous needleleaf forests is relatively high. In addition, the vegetation emissivity decreases with the increase in vegetation water content. Deciduous needleleaf forests had a higher emissivity than tropical rainforests. Research shows that the emissivity of forest elements, such as twigs, branches, and the orientation of leaves, has been measured by ground radiometers at different frequencies [37,38]. The measurements showed that the emissivity is related to the structure and composition of vegetation. Needleleaf forests showed relatively high emissivity, while broadleaf forests showed low emissivity at high frequencies. In winter, due to the influence of deciduous leaves and snowfall, the emissivity of each frequency changes greatly. The emissivity of deciduous needleleaf forests varies strongly with frequency and decreases rapidly at 23.8–89 GHz. This may be because most of the deciduous needleleaf forests in winter are covered by snow, and 89 GHz is very sensitive to surface snow. This is why there was a sharp decrease in the emissivity at 89 GHz in the winter.

### 3.4. Error Sources

The accuracy of derived MWRI surface emissivity is mainly affected by the following factors: the calibration quality of satellite brightness temperatures, the spatial and temporal matching quality of land and atmospheric properties, the parameters obtained from ancillary datasets, the radio-frequency interference, and the microwave sensor channels’ sensitivity to the atmosphere. Errors in brightness temperature calibration can cause some errors in emissivity, such as the noise and bias from nonlinearity corrections. The atmospheric temperature and humidity profiles used in this study are from ERA-Interim reanalysis data with a resolution of 0.75° longitude × 0.75° latitude every 6 h. The errors caused by the temporal and spatial matching between reanalysis data and satellite observation data will affect the emissivity results due to the heterogeneity of atmosphere profiles. Due to the deeper penetration of lower-frequency PMW electromagnetic signals, especially over the arid regions, the variations in land surface temperature and Tb will bring significant errors in surface emissivity retrievals [31,39,40,41]. Past sensitivity analysis [18] reported that a 3 K difference in land surface temperature would result in a difference of a 1% error in surface emissivity estimates, which is about the level of accuracy that is needed in NWP models [42]. The frequency at 10.65 GHz may be affected by the radio-frequency interference. Therefore, the brightness temperature of 10.65 GHz may bring errors to the emissivity retrieval [34]. The microwave sensor channel’s sensitivity to the atmosphere is also one of the factors that will cause error in emissivity retrieval [43]. For those brightness temperatures under a cloudy condition, the estimated emissivity will likely be higher when directly used in the atmosphere profiles from NWP models since it does not include the reliable cloud parameters for radiative transfer calculation. The reduction in surface microwave radiation due to precipitation pixels usually makes the emissivity smaller. The high-frequency channel is more sensitive to the atmosphere. The empirical positive threshold of the brightness temperature difference between low and high frequency for screening rain pixels might have a certain impact on the emissivity results.

## 4. Conclusions

Microwave emissivity values are the signals from observation after removing the effects of land surface temperature and the atmosphere by using an approximated radiation transfer equation. The work presented here provides global land emissivity estimated by brightness temperature and ERA-Interim reanalysis data at the 10.65, 18.7, 23.8, 36.5, and 89 GHz vertical and horizontal polarizations of the FY-3D/MWRI sensor. The estimated emissivity is able to capture the major large-scale features. The low-emissivity areas are due to smaller surface roughness and increased scattering effect. The MPDI has shown that differences between horizontal and vertical polarization signals contain a wealth of information regarding soil moisture and vegetation density. The variations in emissivity and its characteristics among different land covers are also investigated. The emissivity of the deciduous needleleaf forest in summer is almost the greatest among different land cover types. This may be related to factors including vegetation density, vegetation water content, and vegetation composition structure. The error source of emissivity retrieval is also discussed. The land surface temperature, the radio-frequency interference, and the high-frequency channel under cloudy conditions may be the main error sources in this retrieval. This work showed the potential capabilities of providing continuous long term surface microwave emissivity from FY-3 series satellites. The synergy of operational passive microwave sensors may provide comprehensive global emissivity estimates for a better understanding of its spatiotemporal variability and underlying processes.

## Figures and Tables

**Table 1:** The main parameters of MWRI, AMSR2, and GMI sensor. MWRI, microwave radiation imager; AMSR-E, advanced microwave scanning radiometer for earth observing system; GMI, global precipitation measurement microwave imager.

	FY-3, MWRI	GCOM-W, AMSR2	GPM, GMI
Antenna angle of view (°)	53	55	52.8
Equator crossing time (Local time zone)	Ascending: 13:30 Descending: 01:30	Ascending: 13:30 Descending: 01:30	varying
Frequency/Polarizations: footprint (GHz: km × km)	10.65/V, H: 51 × 85 18.7/V, H: 30 × 50 23.8/V, H: 27 × 45 36.5/V, H: 18 × 30 89/V, H: 9 × 15	6.925/V, H: 35 × 62 7.3/V, H: 35 × 62 10.65/V, H: 24 × 42 18.7/V, H: 14 × 22 23.8/V, H: 11 × 19 36.5/V, H: 7 × 12 89/V, H: 3 × 5	10.65/V, H: 19 × 32 18.7/V, H: 11 × 18 23.8/V: 9.2 × 15 36.5/V, H: 8.6 × 14 89/V, H: 4.4 × 7.2 166/V, H: 4.1 × 6.3 183.3/V: 3.8 × 5.8

## Data Availability

Not applicable.
